# *ALK* fusions in the pan-cancer setting: another tumor-agnostic target?

**DOI:** 10.1038/s41698-023-00449-x

**Published:** 2023-09-29

**Authors:** Aditya Shreenivas, Filip Janku, Mohamed A. Gouda, Hui-Zi Chen, Ben George, Shumei Kato, Razelle Kurzrock

**Affiliations:** 1 Medical College of Wisconsin (MCW) Cancer Center, Milwaukee, WI USA; 2 Monte Rosa Therapeutics, Boston, MA USA; 3 Department of Investigational Cancer Therapeutics, The University of Texas MD Anderson Cancer Center, Houston, TX USA; 4 Center for Personalized Cancer Therapy and Division of Hematology and Oncology, Department of Medicine, UC San Diego Moores Cancer Center, La Jolla, CA USA; 5 University of Nebraska, Omaha, NE USA; 6 Worldwide Innovative Network (WIN) for Personalized Cancer Therapy, Chevilly-Larue, France

**Keywords:** Cancer, Cancer genomics

## Abstract

Anaplastic lymphoma kinase (*ALK*) alterations (activating mutations, amplifications, and fusions/rearrangements) occur in ~3.3% of cancers. *ALK* fusions/rearrangements are discerned in >50% of inflammatory myofibroblastic tumors (IMTs) and anaplastic large cell lymphomas (ALCLs), but only in ~0.2% of other cancers outside of non-small cell lung cancer (NSCLC), a rate that may be below the viability threshold of even large-scale treatment trials. Five ALK inhibitors –alectinib, brigatinib, ceritinb, crizotinib, and lorlatinib—are FDA approved for *ALK*-aberrant NSCLCs, and crizotinib is also approved for *ALK*-aberrant IMTs and ALCL, including in children. Herein, we review the pharmacologic tractability of *ALK* alterations, focusing beyond NSCLC. Importantly, the hallmark of approved indications is the presence of *ALK* fusions/rearrangements, and response rates of ~50–85%. Moreover, there are numerous reports of ALK inhibitor activity in multiple solid and hematologic tumors (e.g., histiocytosis, leiomyosarcoma, lymphoma, myeloma, and colorectal, neuroendocrine, ovarian, pancreatic, renal, and thyroid cancer) bearing *ALK* fusions/rearrangements. Many reports used crizotinib or alectinib, but each of the approved ALK inhibitors have shown activity. ALK inhibitor activity is also seen in neuroblastoma, which bear *ALK* mutations (rather than fusions/rearrangements), but response rates are lower (~10–20%). Current data suggests that ALK inhibitors have tissue-agnostic activity in neoplasms bearing *ALK* fusions/rearrangements.

## Introduction

Anaplastic lymphoma kinase (*ALK*) gene alterations are gaining more attention as pan-cancer markers in both solid and hematological malignancies. Activating *ALK* alterations (mutations, amplifications, fusions/rearrangements) are found in various malignancies including, but not limited to, non-small lung cancer (NSCLC), anaplastic large-cell lymphoma (ALCL) (an uncommon, aggressive CD30-positive T-cell lymphoma comprising 0.5% of adult lymphomas and ~10% of non-Hodgkin lymphoma cases in children), inflammatory myofibroblastic tumors (IMT) (rare intermediate-grade neoplasms, generally found in children, which have a high recurrence rate after excision but with low metastatic potential), neuroblastomas, and inflammatory breast cancers. *ALK* genomic alterations are found in ~3.3% of patients with cancers, though *ALK* fusions/rearrangements are less common^1^. In large scale analyses of genomes, *ALK* fusions/rearrangements are detected in ~0.5–0.8% of cancers^1,2^. Among patients with NSCLC, the frequency of *ALK* fusions/rearrangements was over 3%; in contrast, the frequency in non-NSCLC tumors was just ~0.2%. Besides NSCLC, inflammatory myofibroblastic tumor (~50% have ALK fusions/rearrangements) and anaplastic large cell lymphoma (~50–80% having ALK fusions/rearrangements) are the neoplasms most frequently bearing *ALK* fusions. Fusion partners vary widely in non-NSCLC malignancies. Although in NSCLC, most tumors harbored an *EML4*-*ALK* fusion (83.5%), in non-NSCLC malignancies, these constituted the minority (~31%)^1,2^.

**Fig. 1:**
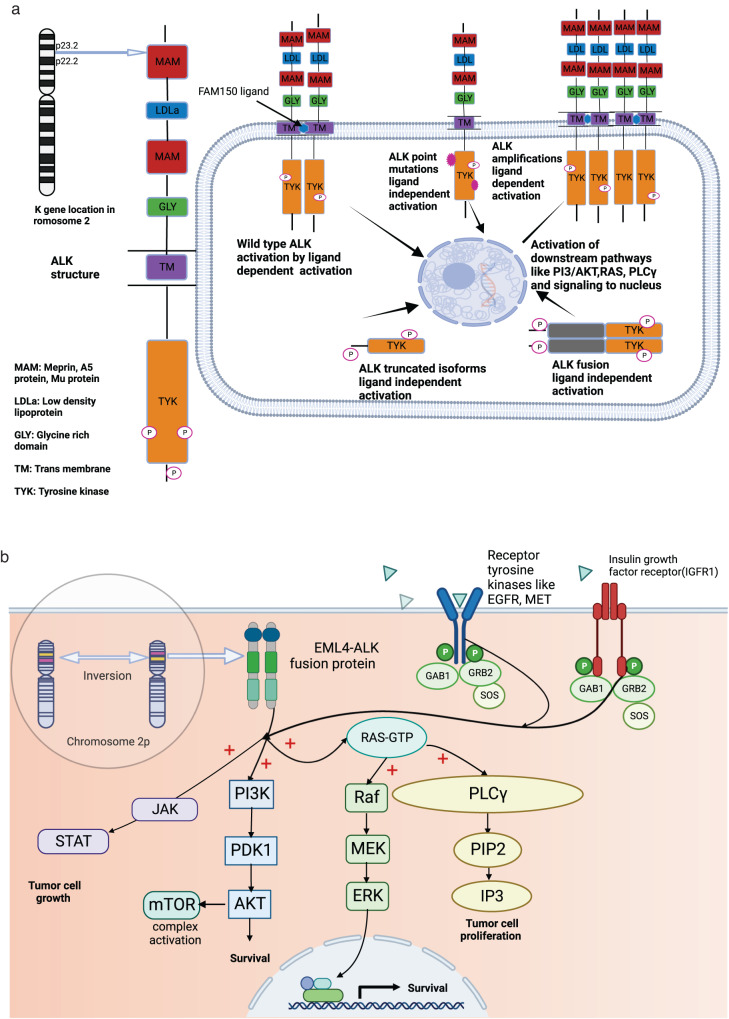
Biology of ALK. **a** ALK gene location, structure, and alterations. The *ALK* gene is located on the short arm of chromosome 2 (2p23). ALK receptor is a member of the insulin receptor superfamily and is a tyrosine kinase enzyme; its structure consists of the extracellular domain (two MAM, one LDLa), and one glycine-rich domain, a connecting transmembrane region, and an intracellular domain (tyrosine kinase domain). ALK receptors (CD246) are activated physiologically by FAM150 ligand binding followed by auto and transphosphorylation of residues, which promotes signaling to the nucleus. Intra tyrosine kinase (TYK) gain of function mutations can lead to ligand-independent downstream pathway activation. Amplified ALK gene sustains downstream signaling in a ligand-dependent manner. Similarly, truncated or isoform ALK gene that loses parts of its extracellular domain and ALK gene fusions can lead to downstream pathway activation, cell cycle progression, proliferation, migration, and angiogenesis. **b** Formation of EML4-ALK4 fusions and activation of downstream pathways. The figure inside the bubble depicts a small inversion within short arm of chromosome 2p, which results in the formation of a fusion gene comprising portions of (*EML4*) and (*ALK*) genes. ALK fusion protein activates downstream signaling pathways, among them, the most relevant and characterized pathways are the (MAPK/ERK), the (JAK-STAT), the (PI3K– Akt), and the (PLCγ) pathways. ALK fusion proteins have a strong oncogenic potential, and it ultimately promotes tumor cell progression. IGF1R and other receptor tyrosine kinase such as EGFR and MET also interact with ALK and lead to activation of downstream pathways. Activation of EGFR and IGF1R sometimes leads to development of bypass resistance pathways. Abbreviations: Akt protein kinase B, ALK Anaplastic lymphoma kinase, EML4 echinoderm microtubule-associated protein-like 4, ERK extracellular signal-regulated kinase, GAB1 growth factor receptor-bound 2 associated binder 1, GRB growth factor receptor-bound, GTP guanosine triphosphate, IP3 inositol triphosphates, JAK Janus kinase, MAPK mitogen-activated protein kinase, mTOR mammalian target of rapamycin, PDK1 pyruvate dehydrogenase kinase 1, PI3K phosphoinositide 3 kinase, PIP2 phosphatidylinositol 4,5-bisphosphate, PKC protein kinase C, PLC-γ phospholipase C γ, RAF rapidly accelerated fibrosarcoma, RAS rat sarcoma virus gene, SOS son of sevenless gene, STAT signal transducer and activator of transcription. Created by Biorender.com.

Normal ALK protein (cluster of differentiation (CD) 246) is a classical tyrosine kinase receptor^3^. It is involved in neuronal and gut development and is transcribed/translated from the normal *ALK* gene located on the short am of chromosome 2 (2p23)^4,5^. Aberrant *ALK* was originally found in an ALCL cell line^6,7^. It was described as a product of t(2;5)(p23;q35) chromosomal translocation involving a nucleophosmin partner (*NPM1)*---(*NPM1-ALK*)^7,8^, discerned in 75–90% of *ALK-*altered ALCL cases, though other *ALK* fusion partners also exist^9,10^. It is now known that *ALK* translocations involve different fusion partners across and within cancers. These translocations facilitate multimerization and autophosphorylation of *ALK*, resulting in a constitutively active tyrosine kinase enzyme that acts as an oncogenic driver (Fig. 1a)^11–19^.

In NSCLC, *ALK* gene alterations act as oncogenic drivers, and occur in ~3–7% of cases^20–22^. One such molecular alteration—an activating fusion of the anaplastic lymphoma kinase (*ALK*) gene with echinoderm microtubule-associated protein-like 4 (*EML4)* gene (normally located on short am of chromosome 2 (2p21))-was first identified by Soda and colleagues^20^ in some NSCLCs. Both the *ALK* and *EML4 g*enes are located on the same short arm of human chromosome 2, but in opposite orientations, and a small inversion involving the two loci, inv (2) (p21p23), results in gene fusion (Fig. 1b)^3,20,23–25^. The fusion gene *EML4-ALK* with its activated tyrosine kinase function induces downstream signaling pathways and promotes cell proliferation and survival. *ALK* gene aberrations in general are more common in the adenocarcinoma histological subtype of NSCLC, in non-smokers, and in young women. Notably, the frequency of *EML4-ALK* fusions commonly found in NSCLC is similar in Asians and Caucasians and the gender difference is rather small but there is a slight female preponderance^26^.

*ALK* translocations in NSCLC are often thought to be mutually exclusive with genomic alterations in the epidermal growth factor receptor (*EGFR*) or the Kirsten rat sarcoma viral oncogene homolog (*KRAS*), though they can occur together^27^.

There are several Food and Drug Administration (FDA) approved ALK inhibitors in the clinic: crizotinib^28^, ceritinib^29^, alectinib^30^, brigatinib^31,32^, and lorlatinib^33^. They are all authorized for the management of NSCLC. The FDA also approved crizotinib for pediatric patients (one year of age and older) and young adults with relapsed or refractory, systemic ALCL that is *ALK*-aberrant^34^. Of note, alectinib is approved for management of relapsed refractory *ALK-*aberrant ALCL in Japan^35^. Recently, the FDA also approved crizotinib for unresectable, recurrent, or refractory *ALK-*aberrant IMT^36^ (Table 1)^28–34,36–39^. The European Medicine Agency (EMA) has also approved multiple ALK inhibitors including crizotinib, ceritinib, alectinib, brigatinib and lorlatinib for treatment of *ALK-*aberrant NSCLC and crizotinib for unresectable ALK-aberrant IMT and ALCL in children and adolescents between the ages of 6 and 18^40^.

**Table 1:** Examples of regulatory approval of ALK inhibitors in multiple cancers.

Drug	FDA approved indication^a^	Date of Approval	Blood brain barrier penetration (low/higher)^b^^37,38^	ALK (IC50) ^c^	Activity	Comment	References
Alectinib	Unresectable or metastatic ALK-positive NSCLC after progression or intolerance to crizotinib	December 2015	Higher	1.9 nM	ORR of 38% and 44% among 87 and 138 patients, respectively, in two single arm trials. NP28761 (NCT01871805) and NP28673 (NCT01801111)	Solid tumor approval	^30^
Alectinib	Unresectable or metastatic ALK-positive NSCLC	November 2017	Higher	1.9 nM	Improvement in PFS: HR of 0.53 (95% CI: 0.38, 0.73; *p* = 0.0001) Among the 43 patients with brain lesions, the CNS ORR was 81%	Solid tumor approval	^30^
Brigatinib	Unresectable or metastatic ALK-positive NSCLC after progression or intolerance to crizotinib	April 2017	Higher	0.37 nM	ORR, ~50%	Solid tumor approval	^31^
Brigatinib	Unresectable or metastatic ALK-positive NSCLC	May 2020	Higher	0.37 nM	ORR, 74%	Solid tumor approval	^32^
Ceritinib	Unresectable or metastatic ALK-positive NSCLC	May 2017	Higher	0.2 nM	Improvement in PFS: HR of 0.55 (95% CI: 0.42, 0.73, *p*-value 0.0001) compared to platinum/pemetrexed. CNS ORR, 57%	Solid tumor approval	^29^
Ceritinib	Unresectable or metastatic ALK-positive NSCLC after progression or intolerance to crizotinib	April 2014	Higher	0.2 nM	ORR, 44%	Solid tumor approval Approval after Phase I trial	^29^ ^39^
Crizotinib	Unresectable, recurrent, or refractory ALK-positive IMT	July 2022	Low	24 nM	ORR, 86%	Solid tumor approval Includes pediatrics	^36^
Crizotinib	Unresectable, recurrent, or refractory ALK-positive ALCL	January 2021	Low	24 nM	ORR, 88% (CR, 81%)	Hematologic malignancy approval Includes pediatrics	^34^
Crizotinib	ALK-positive unresectable/metastatic NSCLC who had previously received one platinum-containing regimen	August 2011 (accelerated) November 2013 (full approval)	Low	24 nM	ORR, ~50–61%	Solid tumor approval	^28^
Lorlatinib	Unresectable or metastatic ALK-positive NSCLC that had progressed on crizotinib and ≥1 other ALK inhibitor for metastatic disease or patients whose disease had progressed on alectinib or ceritinib as the first ALK inhibitor therapy	November 2018	Higher	0.07 nM	ORR, 48% The median response duration was 12.5 months CNS ORR, 60%	Solid tumor approval	^33^
Lorlatinib	Unresectable or metastatic ALK-positive NSCLC	March 2021	Higher	0.07 nM	HR 0.28 (95% CI: 0.19, 0.41; *p* < 0.0001) (lorlatinib versus crizotinib). CNS ORR, 82%	Solid tumor	^33^

Abbreviations: *ALCL* anaplastic large cell lymphoma, *CI* confidence interval, *CNS* central nervous system, *CR* complete remission, *HR* hazard ratio, *IC50* 50% inhibitory concentration, *IMT* inflammatory myofibroblastic tumor, *ORR* objective response rate, *NSCLC* non-small cell lung cancer, *PFS* progression-free survival.

^a^ALK+ infers an *ALK* genomic alteration.

^b^There is overall consensus that crizotinib has low CNS penetration, so we labeled it “low.” In general, other TKI’s like ceritinib, alectinib, brigatinib, and lorlatinib have shown superior CNS activity compared with crizotinib, but to our knowledge, studies have not specifically compared CSF concentrations between them on an ordinal scale, and there is also a discrepancy in CSF concentrations reported in multiple studies, so we thought it would be better to label them as “higher” vs. “high”.

^c^Alk IC50 values were taken from Selleckchem.com. They are on cell-free assay except for crizotinib, which was only reported on cell-based assay.

*ALK* translocations (fusions/rearrangements) and other aberrations such as mutations and amplifications can be detected in multiple solid and hematologic malignancies. *ALK* translocations are particularly vulnerable to pharmacologic targeting. Several trials have therefore addressed possible pan-cancer indications for ALK inhibitors, e.g., Genentech Mypathway trial (NCT02091141) and the phase 2 TAPISTRY platform study (NCT04589845). The FDA has now authorized several tumor-agnostic gene- and immune-targeted agents. For example, agents targeting mismatch repair gene defects, high tumor mutational burden, and aberrant *NTRK, RET*, and *BRAF* are now established tumor-agnostic approvals^41^.

Our review summarizes the biology, diagnostic approach, therapeutic options, resistance mechanisms, and novel strategies for management of *ALK*-aberrant malignancies, with a particular emphasis on cancers beyond NSCLC, and the potential for viewing aberrant *ALK* as a tumor-agnostic target (including for both solid tumors and hematologic malignancies and for both common and uncommon cancers across the age spectrum). Tumor-agnostic indications may be especially important for rare cancer types, which represent an unmet need in oncology. We also present an illustrative case of an ultra-rare neoplasm---Erdheim Chester Disease (non-Langerhans histiocytosis) ---with an *ALK* fusion, and brain involvement, remarkably responsive to an ALK inhibitor.

## Biology of normal and aberrant Alk

### ALK (CD246) structure and function

*ALK*, located on the short arm of chromosome 2, is a member of the insulin receptor protein−tyrosine kinase superfamily (Fig. 1a)^11–19^. These transmembrane tyrosine kinase enzymes regulate cellular growth and trigger neoplastic transformation. Like other receptor tyrosine kinases, ALK undergoes a ligand-induced activation in extracellular space leading to its homo-dimerization or hetero-dimerization. FAM150A (ALKAL1 or Augmentor **α**) and FAM150B (ALKAL2 or Augmentor β) are group of peptides that act as ligands and activate ALK receptor tyrosine kinases^17,18^. The dimerization of ALK receptor tyrosine kinase results in the trans-phosphorylation of specific tyrosine residues within the cytoplasmic domain of ALK, which then leads to more tyrosine residues being phosphorylated on the same receptor tyrosine kinase. Activated ALK then phosphorylates tyrosine residues on its substrate proteins, which finally activates downstream oncogenic signaling pathways^23^ .

At the structural level, the human *ALK* gene consists of two meprin A 5 proteins, receptor protein tyrosine phosphatase μ regions (MAM), glycine-rich domains (GR), a low-density lipoprotein motif (LDLa), and an intracellular domain of tyrosine kinase (Fig. 1a)^11–19^.The ALK protein is a receptor tyrosine kinase that encompasses 26 exons that encode 1620 amino acid proteins of which about 180 kDa is glycosylated. ALK consists of an intracellular tyrosine kinase domain with three-tyrosine motifs (Tyr1282, Tyr1283 and Tyr1278) that act as auto-phosphorylation site for regulating kinase activity, extracellular ligand-binding domain and a transmembrane domain^11,42^.

### *ALK* alteration types and biology

ALK proteins are commonly overexpressed or aberrantly expressed in tumors due to *ALK* gene rearrangements or fusions, copy number gains or gene amplification, and activating kinase mutations. Overall, ~3.3% of cancers across the malignancy spectrum harbor *ALK* alterations, with *ALK* fusions detected in around 0.5–0.8% of all cancers (~0.2% of cancers outside of NSCLC)^1,2^. Among them, the *EML4-ALK* fusion is the most common^1^. In ALCL, the most common rearrangement results in the *NPM1-ALK* fusion^16^. Other common alterations in *ALK* are activating kinase point mutations and *ALK* amplifications, observed in around 2.8% to 3.0%^1,43^ and 0.10% of all cancers respectively^1^.

The constitutive kinase activity of *ALK* fusions may arise from self-dimerization through N-terminal oligomerization domains, leading to auto- and transphosphorylation of ALK. Other oncogenic mechanisms leading to aberrant ALK activity include point mutations in the kinase domain (that enhance kinase enzymatic function) and gene amplification; however, not all mutations in *ALK* lead to ligand-independent or even kinase-active protein and some may merely represent passenger mutations. *ALK* rearrangements/fusions are particularly important in cancer because they are pharmacologically tractable. A variety of fusions have been described (Table 2)^2,7,16,20,44–66^.

**Table 2:** Examples of *ALK* translocations, chromosomal locations, and relative frequencies in solid and hematologic malignancies.

ALK partners	Tumor type comment	Chromosomal alterations	ALK fusion approximate frequency (%) in tumor	References
	Solid Tumors			
ATIC	IMT	inv (2) (p23q35)	50–55% of all IMT	^44^
A2M	IMT	t (2;12) (p23; p13)		^45^
CLTC	IMT	t (2;17) (p23; q23)		^16^
RANBP2	IMT	inv (2) (p23q11-13)		^16,66^
TPM3 and TPM4	IMT	t(1;2) (q21;p23) and t(2;19)(p23;p13)		^46^
PPFIBP1	IMT	t (2;12) (p23; p11)		^16^
SQSTM1	NSCLC	t (2;5) (p23.1; q35.3)	3–7% of all NSCLC	^16^
CLTC	NSCLC	t (2;17) (p23; q23)		^2^
HIP1	NSCLC	t (2;7) (p23; q11.23)		^2,16^
DCTN1	NSCLC	inv (2) (p13p23)		^2^
A2M	NSCLC	t (2;12) (p23; p13)		^16^
TPR	NSCLC	t (1;2) (q31.1; p23)		^16^
EML4	NSCLC Most common ALK partner in NSCLC	inv (2) (p21p23)		^20^
KIF5B	NSCLC	t (2;10) (p23; p11)		^2,16^
SPTBN1	CRC	t (2) (p16.2; p23)	<1% of all CRC	^47^
CAD	CRC	inv (2) (p23; p22)		^48^
EML4	CRC	inv (2) (p21p23)		^58^
STRN	CRC	t (2) (p23; p22.2)		^49^
VCL	RCC	t (2;10) (p23; q22)	<1% of all RCC	^16^
STRN	RCC	t (2) (p23; p22.2)		^50^
TPM3	RCC	t (1;2) (q21; p23)		^16^
EML4	RCC	inv (2) (p21p23)		^16^
DCTN1	PDAC	inv (2) (p13p23)	N/A	^64^
STRN	CCA	t (2) (p23; p22.2)	N/A	^51^
EML4	BC	inv (2) (p21p23)	N/A	^52^
TPM4	ESCC	t (2;19) (p23; p13)	N/A	^53^
FN1	OC	inv (2) (p23q34)	N/A	^57^
STRN	MPM	t (2) (p23; p22.2)	N/A	^54^
STRN	TC	t (2) (p23; p22.2)	<1% of all TC	^55^
DCTN1	TC	inv (2) (p13p23)		^16^
FN1	LMS	inv (2) (p23q34)	N/A	^56^
	Hematological malignancies			
TFG	ALCL	t (2;3) (p23; q21)		^59^
RNF213/ALO17	ALCL	t (2;17) (p23; q25)		^16^
NPM1	ALCL	t (2;5) (p23; q35)	~80% of pediatric and ~50% of adult ALCL harbor NPM1-ALK	^7,16^
CLTC	ALCL	t (2;17) (p23; q23)		^60^
MSN	ALCL	t (2; X) (p32; q11-12)		^16^
TPM3	ALCL	t (1;2) (q25; p23)		^16^
TPM4	ALCL	t (2;19) (p23; p13)		^16^
EML4	DLBCL	inv (2) (p23; q21)	<1% of all DLBCL	^61^
SEC31A	DLBCL	t (2;4) (p24; q21)		^62^
CLTC	DLBCL	t (2;17) (p23; q23)		^65^
RANBP2	AML	Inv (2) (p23; q13)	N/A	^63^
KIF5B	Histiocytosis	t (2;10) (p23; p11)	N/A	^2^

Abbreviations: *A2M* α-2-macroglobulin, *ALCL* anaplastic large cell lymphoma, *AML* acute myeloid leukemia, *ATIC* 5-aminoimidazole-4-carboxamide ribonucleotide formyltransferase, *BC* breast cancer, *CAD* carbamoyl-phosphate synthetase 2, aspartate transcarbamylase, and dihydroorotase, *CRC* colorectal cancer, *CCA* cholangiocarcinoma, *CLTC* clathrin heavy chain, *DCTN1* dynactin, *DLBCL* diffuse large B cell lymphoma, *EML4* echinoderm microtubule-associated protein like-4, *ESCC* esophageal squamous cell carcinoma, *FN1* fibronectin 1, *HIP1* huntingtin-interacting protein 1, *IMT* inflammatory myofibroblastic tumor, *KIF5B* kinesin family member 5B, *LMS* leiomyosarcoma, *MPM* malignant peritoneal mesothelioma, *MSN* moesin, *NA* not available, *NPM1* nucleophosmin1, *NSCLC* non-small cell lung cancer, *OC* ovarian carcinoma, *PDAC* pancreatic ductal carcinoma, *PPP1CB* PP1-beta-catalytic subunit, *RANBP2* RAN binding protein 2, *RCC* renal cell carcinoma, *RNF213* ring finger protein 213, *SEC31A* SEC31 homolog A, *SPTBN1* spectrin beta non-erythrocytic 1, *SQSTM1* sequestosome 1, *STRN* striatin, *TC* thyroid carcinoma, *TFG* TRK-fused gene, *TPM3* tropomyosin 3, *TPM4* tropomyosin 4, *TPR* translocated promoter region, *VCL* vinculin.

NSCLC is one of the cancers in which *ALK* gene fusions/rearrangements have been especially well recognized, with ~3–7% of cases affected^20–22^. The most common rearrangements in NSCLC result from an inter-chromosomal inversion in the short arm of chromosome 2, which creates a fusion between the 5’ portion of the *EML4* gene and the 3’ portions of the *ALK* gene Inv (2) (p21p23) (Fig. 1b)^3,20,23–25^.

In vitro studies utilizing *NPM1-ALK* and *EML4-ALK*-based model systems demonstrate that dysregulation of ALK activity via fusion leads to activation of four key downstream oncogenic signaling pathways: Janus kinase - signal transducers and activators of transcription (JAK-STAT)^67^, mitogen-activated protein kinase/extracellular signaling regulated kinase) (MAPK/ERK)^68^, phospholipase C gamma (PLCγ)^69^ and phosphatidylinositol-3-kinase – protein kinase B) (PI3K-Akt)^69^. These pathways are important in cell cycle progression, proliferation, apoptosis, angiogenesis, and cell survival (Fig. 1b)^3,20,23–25^.

## *Alk* alterations in solid and hematologic malignancies

The solid tumor best known for *ALK* alterations is NSCLC. However, *ALK* is also altered in a wide array of other solid tumors, as well as in hematologic cancers, with important clinical implications. *ALK* alterations in malignancies include fusions, mutations, amplification, and overexpression (Table 3)^1,2,20–22,34,36,52,63,66,70–90^.

**Table 3:** Selected examples of tumors with ALK alterations and their clinical implications.

Examples of diseases	ALK alterations and their frequency	Comments	References
All cancers	~3.1% of cancers have ALK alterations.^a^ ~0.2% of all cancer types have ALK fusions/rearrangements	Alterations other than fusions may include mutations, amplifications and ALK positivity by immunohistochemistry. ALK mutations are found in about ~3% of various cancer types	^1,2^
Solid tumors			
Breast carcinoma (including inflammatory breast cancers)	ALK gene amplifications are found in ~13% of all breast cancers, more frequently triple negative breast cancers or inflammatory breast cancer^70^ 75–80% of inflammatory breast cancers can have ALK copy number gains and amplifications^77^ EML4-ALK fusions are found in ~2.4% of breast cancers^52^^b^ ALK mutations were observed in ~1.8% of cases of breast carcinomas in AACR Genie database.	ALK protein positivity may not be present in many inflammatory breast cancers despite ALK high copy number gains and amplifications^88^ Data on responsiveness to ALK inhibitors in ALK-amplified breast cancer is not well described in the literature.	^1,52,70,77,88^
GBM	ALK amplifications are present in around 0.2% of all of glioblastoma cases. ALK Mutations were observed in ~2.7% cases of GBM in the AACR Genie database. PPP1CB-ALK fusions were observed in ~43% (7/16) and ALK amplification was observed in 31% (5/16) of ALK-expressing pediatric glioblastoma cases^89^	ALK overexpression by IHC may be present in over 40% of cases, but fusions are uncommon.	^1,76,89^
IMTs	ALK fusions are found in ~50% of IMT patients. The majority of ALK alterations seen in IMT are fusions. Multiple fusion partners to *ALK* occur some of them are included in (Table 2)	ALK expression in these tumors can be associated with better prognosis. When RANBP2 is the fusion partner to ALK, it is often associated with epithelioid inflammatory myofibroblastic sarcoma, an aggressive subtype of IMT^66^ The FDA approved crizotinib for unresectable, recurrent, or refractory ALK-aberrant inflammatory myofibroblastic tumors.	^1,36,66,79–81^
Melanoma	Spitz nevi and spitzoid melanomas are associated with ALK fusions in around 8–15% cases. ALK Mutations were observed in ~7.5% of cases of melanoma in AACR Genie database.		^1,2,75,78^
NSCLC	ALK fusions, ~3–7% of NSCLC ALK amplification were observed in~ 0.02% and ALK Mutations seen ~3.6% NSCLC cases of AACR genie data base.	EML4-ALK is the most common ALK fusion. Multiple ALK inhibitors approved (see Table 1)	^1,20–22^
Neuroblastomas	8–16% of newly diagnosed neuroblastoma have somatic ALK alterations. ALK mutations were observed in ~12.4% of cases of neuroblastoma in AACR Genie database. Around ~1–2% of neuroblastoma cases are inherited (autosomal dominant) and almost 50% of them have germline gain of function ALK mutation. ~1–3% of neuroblastoma have ALK amplification.	ALK-mutant versus wild-type neuroblastoma have inferior survival, aggressive disease. Mutations in three positions in kinase domain—R1275, F1174, and F1245—account for around~85% of ALK mutations in neuroblastomas; R1275Q is the most common mutation, present in 45% of familial cases and a third of sporadic cases, whereas F1174 and F1245 mutants are exclusively found in sporadic disease at frequencies of around 30% and 12%, respectively. ALK can be co-amplified or co-mutated with MYCN, consistent with the proximity of these genes at 2p23-24 which is associated with aggressive prognosis.	^1,82–84^
Prostate	ALK gene truncations were found in 6.4% of cases in some studies. ALK protein overexpression through IHC found in 9% cases of prostate cancer	Rare SLC45A3-ALK fusion and ALK F1174C mutation has been observed	^1,85–87^
Hematologic malignancies			
ALCL	~80% of pediatric and ~50% of adult ALCL harbor NPM1-ALK ALK mutations were observed in ~2.8% cases of ALCL in the AACR Genie database.	The FDA approved crizotinib for pediatric patients (one year of age and older) and young adults with relapsed or refractory, systemic anaplastic large cell lymphoma (ALCL) that is ALK-aberrant.	^1,34,71,72^
Histiocytosis	In a study of 39 ALK positive histiocytosis, 37 had ALK rearrangements, 27 with KIF5B-ALK fusion	ALK-fusion histiocytosis is a rare subtype of histiocytic neoplasm. KIF5B is the most common ALK fusion partner. Others observed DCTN1, TPM3, EML4 and TFG fusions with ALK	^74^
Leukemia		RANBP2-ALK fusion has been reported in an AML case^63^ ALK mutations in 2/185 (<1%) cases of leukemias)	^63,90^
Multiple myeloma	TFG-ALK fusion has been reported in non-secretory multiple myeloma	TFG-ALK fusion has been reported in non-secretory multiple myeloma	^73^

Abbreviations: *ALCL* anaplastic large cell lymphoma, *ALL* acute lymphoblastic leukemia, *AML* acute myelogenous leukemia, *EML4* echinoderm microtubule-associated protein like-4, *GBM* glioblastoma multiforme, *IHC* immunohistochemistry, *IMT* inflammatory myofibroblastic tumor, *KIF5B* kinesin family member 5B, *NPM1* nucleophosmin1, *NSCLC* non-small cell lung cancer, *PPP1CB* protein phosphatase 1 catalytic subunit beta, *RANBP2* RAN binding protein 2, *TFG* TRK-fused gene, *TPM3* tropomyosin 3.

^a^ALK alterations in AACR genie database included rearrangements, mutations and amplification.

^b^The incidence rate of EML4-ALK fusion in this study was reported by using relatively older technology (exon array profiling) and no further studies have supported this finding.

### *ALK* gene alterations in NSCLC

*ALK* gene alterations are found in ~3–7% of cases^20–22^. *ALK* gene rearrangements (leading to fusions), as well as mutations and amplifications can be discerned. Desai and colleagues analyzed 11,107 tumor samples from 10,082 patients of lung adenocarcinoma from AACR Genie data base^91^ and found 584 (5%) samples with *ALK* gene alterations: 354 missense mutations (60.6%); 265 cases with fusions (45.4%); 51 with truncating mutations (8.7%); and 1 case with in-frame mutation (0.17%).

*ALK* gene rearrangement can manifest in the form of a translocation with another partner gene leading to formation of a fusion oncogene. These fusions often arise from fusion of the 3′ end of the *ALK* gene (exons 20–29) with the 5′ portion of a different gene^92^. For example, in some NSCLC cases, inversion rearrangement from inv(2) (p21;p23) results in *EML4* replacing the extracellular and intramembranous parts of *ALK* and fusing with its juxtamembrane domain leading to the formation of *EML4-ALK* fusion oncogene^20^.

*EML4* is the most common fusion partner in NSCLC, found most NSCLCs with *ALK* fusions^2^. Still, some studies have identified more than a dozen *ALK* gene rearrangements involving various *EML4-ALK fusion* breakpoints^93^.. Apart from *EML4*, other fusion partners seen in NSCLC include *SQSTM1* (sequestosome), *DCTN1*(dynactin), *HIP1*(huntington intercating protein 1) *and KIF5B* (kinesin family member 5 B)^16,91^.

### *ALK* gene alterations in cancers beyond NSCLC

*ALK* gene alterations such as gene fusions, mutations and amplification have also been described in multiple solid tumors apart from NSCLC including, but not limited to, inflammatory myelofibrotic tumor (IMT), neuroblastoma, esophageal carcinoma, renal medullary carcinoma, breast carcinoma, colorectal carcinoma, serous ovarian carcinoma, and thyroid carcinoma. *STRN* (striatin) and *NPM1* (nucleophosmin) are the most common fusion partners of *ALK* in non-NSCLC tumors^2^.

IMT are rare mesenchymal tumors that are mostly seen in the pediatric and adolescent populations. Importantly, they are associated with *ALK* gene rearrangement in around 50% of cases^79,80^. ALK overexpressing IMT’s may have a better prognosis than ALK non-expressing tumors^81^. About half of IMT’s are associated with rearrangements involving the *ALK* gene locus on chromosome 2p23 juxtaposed to several different translocation partners: *TPM3* and *TPM4 (tropomyosin 3 and 4)*, *CLTC 1 (clarithin heavy chain 1)* and *RANBP2 (RAN binding protein 2)*^16^. Among them, *RANBP2* is often associated with epithelioid inflammatory myofibroblastic sarcoma, which is an aggressive subset of IMT^66^.

As mentioned*, ALK* fusions are also observed in renal cell carcinoma, thyroid carcinoma, pancreatic adenocarcinoma^94^, spitzoid melanocytic tumors^75^, salivary gland cancers^95^, and colorectal cancers^16^ (Table 2)^2,7,16,20,44–66^. Point mutations in *ALK* also occur and can activate the downstream pathways in a ligand-independent manner. Gain-of-function point mutations usually occur within the kinase domain of ALK receptor tyrosine kinase enzyme. Some *ALK* mutations have also been identified in neuroblastomas and can either be germline or somatic^12,96^. Types and frequencies of different *ALK* alterations seen in cancer have been tabulated in (Table 3)^1,2,20–22,34,36,52,63,66,70–90^.

*ALK* alterations are present in 8–16% of newly diagnosed patients with neuroblastoma^82–84^. Around ~1–2% of neuroblastoma cases are inherited (autosomal dominant) and ~50% of them have germline gain-of-function *ALK* mutation^83^. The majority of *ALK*-altered neuroblastoma are associated with sporadically acquired somatic mutations. Among the somatic mutations, single-nucleotide variants of ALK tyrosine kinase domain at loci R1275, F1174, and F1245 are the most common and seen in ~85% of all cases with alterations^82,83^. *ALK-*mutant neuroblastomas have inferior survival compared with those with *ALK* wild-type (WT) tumors^83,97^. A*LK* F1174L mutated neuroblastoma is thought to be an aggressive disease phenotype, which confers resistance to crizotinib^82,83^. Furthermore, co-expression of *ALK* F1174L and *MYCN* amplification is associated with poor prognosis in neuroblastoma^82,83^.

Another mechanism of gene alteration is via *ALK* gene locus amplification. This process was first studied in neuroblastoma cell lines^13^. These amplifications often result in ligand-dependent activation of downstream ALK signaling pathway. They are found in ~4% of high-risk neuroblastomas and confer a poor prognosis^14,98^. *ALK* fusions and amplifications have also been reported in glioblastoma. ALK can be overexpressed in 43%–70% of glioblastoma cases and may have biologic relevance^99–102^. *PPP1CB*-*ALK* fusions were observed in~43% (7/16) and ALK amplification in 31% (5/16) of *ALK*-expressing pediatric glioblastoma cases^89^; ALK translocations are rare in adult GBM.

*ALK* gene amplifications are found in ~13% of breast cancers^70^ and ~3% of colorectal cancers^103^. There may be a high frequency (~75%) of *ALK* gene amplifications in inflammatory breast cancer^104^. *ALK* copy number gains and amplifications are also seen in some types of rhabdomyosarcomas^105^.

Truncated mutations are formed by elimination of the extracellular domain of *ALK* gene which lead to activation of downstream pathways and have been identified in neuroblastoma, squamous cell carcinoma of the skin and melanomas^15,78,106^. For example, In-frame deletion of exons 1 through 5 (Δ 1–5) and exons 4 through 11 (Δ 4–11) in *ALK* gene are seen in neuroblastoma cell lines^15^ whereas *ALK* Δ 2–17 are usually found in synovial sarcoma cell lines^107^. Additionally, *ALK* intra-kinase domain mutations have also been described; they can be acquired after exposure to certain ALK inhibitors and may perpetrate resistance.

*ALK* gene rearrangements/fusions are commonly found in hematological malignancies such as ALCL, which is a moderately aggressive T-cell lymphoma. *ALK* fusions play a vital role in *ALK-*altered ALCL pathogenesis. Earlier studies have shown that ALK*-*expressing ALCL have a much better 5-year overall survival rate (70–90% vs 15–62%) compared to non-ALK*-*expressing ALCL^16,108^. The *NPM1-ALK* fusion is found in >80% of pediatric ALCL and in >50% of adult ALCL cases. As described earlier, this results from the translocation of the *NPM1* gene at 5q35, which encodes a nucleolar protein involved in transporting ribonucleoproteins from the cytoplasm to the nucleus, to the *ALK* gene at 2p23, encoding a tyrosine kinase receptor. Non-*NPM1* fusion partners (e.g., *TFG, TPM3, TPM4, CLTC 1*, ALO17 and MSN) are also found in some *ALK-*altered ALCL. Among them, *TPM3* is the most common in ALCL^2,16^.

*ALK* fusions are also found in DLBCL but also in differentiated B cell lymphomas, some leukemias, myelomas and histiocytosis^2^. *ALK f*usions in DLBCL are associated with poor prognosis. While most of them are associated with *CLTC-ALK* fusion, other *ALK* fusion partners such as *NPM1* and *EML4* are also seen^16,65^.

*ALK* alterations are rare in leukemia. *RANB2-ALK* fusions have been detected in three pediatric cases of atypical myeloproliferative leukemia^109^ and an adult patient with acute myelomonocytic leukemia^63^. A few cases harboring *NPM1-ALK* fusions have been reported in B cell acute lymphoblastic leukemia^2^. Oncogenic point mutations (A348D and F856S) have also been discovered in an acute myelogenous leukemia (AML) and a B-acute lymphoblastic leukemia (B-ALL) patient, respectively^90^. Unlike point mutations seen in neuroblastoma which are mainly found in kinase domain, the mutations found in leukemia were in the extracellular domain. Rare oncogenic deletion mutations of *ALK* have also been described in ALCL cells^110^.

## Alk-targeted therapies

There are now several drugs that have achieved regulatory approval for *ALK*-altered tumors: alectinib, brigatinib, crizotinib, ceritinib, and lorlatinib. The majority of approvals are for NSCLC. However, the FDA has also approved ALK inhibitors such as crizotinib for IMT and for the hematologic malignancy ALCL (Table 1)^28–34,36–39^.

### ALK inhibitors used in treatment of ALK-altered NSCLC

Alterations in the *ALK* gene sensitize NSCLC to ALK inhibitors, which bind to receptor tyrosine kinases and inhibit downstream signaling pathways. To date, five ALK inhibitors have received approval from the Food and Drug Administration (FDA) for *ALK*-altered NSCLC treatment: first-generation crizotinib, second-generation (ceritinib, alectinib, and brigatinib) and third generation ALK inhibitors (lorlatinib) based on their activity in the clinic (Supplementary Table 1)^111–127^.

Crizotinib was the first oral tyrosine kinase inhibitor approved for the treatment of NSCLC. Its targets include ALK, ROS1 and MET alterations. Its FDA approval in 2011 was based on landmark studies like PROFILE 1005^111^. These results led to the development of two other phase III studies (PROFILE 1007^114^ and PROFILE 1014^113^), which confirmed the efficacy of crizotinib and its superiority over platinum-based chemotherapy regimens in both second and first-line settings respectively. Crizotinib unfortunately has poor blood brain barrier penetrance^37^ and patients on crizotinib often develop resistant mutations. This led to the development of second and third generation drugs.

Ceritinib was the second oral ALK inhibitor that received FDA approval. The approval was in 2014 for patients with *ALK*-altered NSCLCs that progressed or were intolerant to crizotinib and as first-line in 2017. Ceritinib is 20 times as potent as crizotinib with activity against several *ALK* mutations such as L1196M G1 269 EA IM 1171T, and S1206Y. The initial approval of ceritinib was based on ASCEND 1^119^ and 2^122^ trials. It further received first-line treatment approval in treatment-naïve patients based on results from ASCEND 4 study^118^. Ceritinib inhibits autophosphorylation of ALK and targets IGF1R, and ROS1 alterations. Its ability to inhibit IGF1R may contribute to its activity in crizotinib-resistant cases^24,25^. Unlike crizotinib, other second and third generation ALK inhibitors have good blood-brain barrier penetration^38^ (Table 1)^28–34,36–39^.

The third ALK inhibitor to be developed was alectinib; its targets include both *ALK* and *RET* alterations. Alectinib is a 5-fold stronger ALK inhibitor compared to crizotinib and has good central nervous system activity. It does not have a substrate of P-glycoprotein, which is a key efflux transporter located at the blood-brain barrier. It has been FDA approved in *ALK*-altered NSCLC with or without previous treatments on crizotinib. This approval in 2015 was based on results of two single arm Phase II trials^115,128^. Alectinib is active in tumors harboring C1156Y, G126 9A, S1206Y and L1152R mutations but not G1202R. The superiority of alectinib over crizotinib was reported in the ALEX trial^120^, which compared alectinib to crizotinib in treatment-naïve patients with ALK-altered NSCLCs.

Similarly, brigatinib was another ALK inhibitor which received FDA approval based on results of ALTA trials. It is a second-generation tyrosine kinase inhibitor (TKI) that impacts the products of *ALK* and *ROS1* fusions and interestingly also mutant *EGFR L858R*. It also has inhibitory activity against several acquired *ALK* mutations but questionable activity against *G1202R*. US FDA granted accelerated approval to brigatinib for treatment of individuals with advanced *ALK*-altered NSCLC that had progressed or were intolerant to crizotinib; approval was based on results of phase II ALTA^124^ and in first-line setting based on results from Phase III ALTA-1L trial^121^.

Finally, lorlatinib is a third generation ALK inhibitor that targets *ALK* and *ROS1*. It is specifically designed to target resistant mutations associated with first- and second-generation ALK inhibitors including the *G1202R* mutation. It first attained FDA approval in 2018 in clinically advanced *ALK*-altered NSCLC patients that had progressed on first- and second-generation ALK inhibitors such crizotinib/ceritinib/alectinib based on results of a phase II study^112^. Additionally, impressive results of the recently concluded CROWN study^117^ have led to its FDA approval in the first-line setting. Lorlatinib is also associated with excellent intracranial activity^129^.

### ALK Inhibitors in solid tumors (beyond NSCLC) and in hematologic malignancies

ALK inhibitors have shown activity in a range of solid tumors and hematologic malignances and have achieved approval in the rare solid tumor IMT and in the hematologic malignancy ALCL, in addition to the NSCLC approval (Table 1)^28–34,36–39^ and (Table 4)^35,48,73,74,85,94,126,130–149^.

**Table 4:** Examples of studies and cases of targeted therapies in ALK-altered cancers (other than NSCLC) (See Supplemental Table 1 for NSCLC studies).

Tumor characteristics (No. of patients) Study Title	ALK alteration	Type/Phase of study	Drug	CR/PR (*N*/total *N* (%))	Comment	References/CT.gov ID number if ongoing
ALCL (*N* = 16) A8081013 (NCT01121588) PROFILE 1013	NPM1-ALK fusion	Phase IB study in non-NSCLC tumor	Crizotinib	9/16 (56%)		^132^
ALCL (*N* = 26) ADVL0912 (NCT00939770)	NPM1-ALK fusion	Phase I/II	Crizotinib	ALCL165 group: 5/6 (83%) ALCL280 group: 18/20 (90%)	ALCL cases treated at doses of 165 mg/m2 (ALCL165) and 280 (ALCL280) mg/m2	^130^
ALCL (*N* = 10 in ALCL cohort)	NPM1-ALK fusion	Phase II	Alectinib	8/10 (80%)		^35^
Colon cancer (*N* = 1)	CAD-ALK fusion	Case report	Entrectinib ALKA-372-001 phase I study	1/1(100%)	DOR 4+ months	^48^
Colon cancer (*N* = 1)	EML4-ALK fusion (E21; A20) and	Case report	Crizotinib Alectinib Lorlatinib	1/1(100%)	Patient was treated with crizotinb, and had a PR, Developed leptomeningeal disease switched to alectinib for few weeks and then lorlatinib; had PR with latter and DOR was 11 months	^141^
DLBCL (*N* = 2)	ALK fusion partner unknown	Case series	Crizotinib	1/2 (50%)		^133^
Erdheim-Chester disease (non-Langherhans histicocytosis) (*N* = 1)	ALK-KIF4B	Case report	Alectinib	1/1(100%)	DOR 6+ months	Included in this manuscript
Glioma, high grade (*N* = 1)	SPECC1L–ALK fusion	Case report	Lorlatinib	1/1 (100%) 3 y/o boy		^146^
Histiocytosis with ALK fusions (*N* = 11)	KIF5B-ALK fusion (*N* = 10) DCTN1-ALK fusion (*N* = 1)	Retrospective study	Crizotinib Alectinib Brigatinib Lorlatinib Ceritinib	11/11(100%)	Median time on ALK inhibitors 16 months (range 3-43 months)	^74^
Inflammatory myofibroblastic sarcoma (EIMS) (epithelioid) (*N* = 1)	PRRC2B-ALK fusion	Case report	Crizotinib Alectinib Ceritinib Lorlatinib	1/1 (100%)	Patient was treated with four sequential ALK inhibitors and had PR to first three ALK Inhibitors	^142^
IMT (*N* = 14 pediatric patients.) (NCT00939770)	NPM-ALK fusion	Phase I/II	Crizotinib	12/14 (86%)	Circulating tumor-derived NPM-ALK transcript decreased with response.	^130^
IMT (*N* = 2)	Usually bear ALK fusions	Case series	Brigatinib	1/2 (50%)		^134^
IMT of head and neck (*N* = 1	SQSTM1-ALK fusion gene	Case report	Alectinib	1/1 (100%)	DOR 17+ months	^135^
Mesothelioma (peritoneal)	STRN-ALK Fusion	Case report	Ceritinib	1/1(100%)	DOR 3+mths	^144^
Multiple myeloma, non-secretory (*N* = 1) Myeloma	TFG-ALK fusion	Case report	Alectinib	1/1(100%)	DOR 24+months	^73^
Neuroblastoma (*N* = 1)	ALK mutation at exon 24 F1245C	Case report	Alectinib	1/1 (100 %)	DOR 12+ months	^136^
Neuroblastoma (*N* = 28) A New Approaches to Neuroblastoma Consortium study	ALK mutations/amplifications	Phase I	Lorlatinib	In (2–17) age group, 1/18 (5%) In (15–50) age group, 4/10 (40%)		^148^
Ovarian Cancer (Refractory high-grade serous) [*N* = 1]	EML4-ALK Fusion	Case report	Alectinib	1/1(100%)		^147^
Pancreatic adenocarcinoma (*N* = 1)	PPFIBP1-ALK fusion	Case report	Alectinib/ Lorlatinib		Minor response with alectinib lasted for 5 months.Pt. achieved stable disease for 2 months on lorlatinb	^94^
Pancreatic adenocarcinoma (*N* = 1)	EML4-ALK fusion	Case report	Crizotinib Alectinib	1/1 (100%) to both crizotinib and alectinib	PFS with crizotinib, 8 months PFS with alectinib 10 months	^137^
Pancreatic neuroendocrine cancer (*N* = 1)	KANK1-ALK Fusion	Case report	Lorlatinb	1/1 (100%)	DOR 4+ months	^149^
Prostate cancer (*N* = 1)	ALK F1174C-activating point mutation	Case	Alectinib	Stable disease >6mths		^85^
Renal cell carcinoma (*N* = 1)	VCL-ALK fusion	Case report	Entrectinib ALKA-372-001 and STARTRK-1 phase I study	1/1(100%)		^126^
Thyroid cancer, medullary (*N* = 1)	CCDC6-ALK fusion	Case report	Crizotinib/Alectinib	1/1(100%)		^139^
Thyroid cancer, anaplastic (*N* = 1)	STRN-ALK fusion	Case report	Ceritinib /Brigatinib	1/1(100%)	DOR 15+ months	^138^
Studies with multiple tumor types						
ALK-altered tumors such as ALCL [*N* = 1], IMT [*N* = 4], GBM, *N* = 12] and others [*N* = 5]) ≥1 prior systemic therapy ASCEND 10 study. (NCT02465528)	Type of alterations not reported. But IMT and ALCL usually bear fusions..GBM generally have non-fusion ALK alterations such as overexpression)	Phase II	Ceritinib	ALCL 1/1(100%) IMT 3/4 (75%) Glioblastoma 0/12 (0%)		^140^
Neuroblastoma (*N* = 11) Children’s Oncology Group consortium study of 79 pts. (including neuroblastoma, IMT, NSCLC and ALCL) (NCT00939770)	ALK rearrangements seen in ALCL and IMT ALK mutations seen in neuroblastoma. Arg1275 Gln (patient with CR)	Phase I/II	Crizotinib	Neuroblastoma 1/11(9%)		^131^
NCI match subgroup F Colorectal carcinoma [*N* = 2] Carcinoma of unknown primary [*N* = 1] Leiomyosarcoma [*N* = 1] (NCT02465060)	EML4-ALK STRN-ALK ACTG2-ALK fusions	Phase II	Crizotinib	2/4 (50%)	CR in leiomyosarcoma and PR in colorectal	^145^
Ongoing studies						
Plasmablastic ALK-altered large B cell lymphoma.		Phase II	Belantomab Mafodotin			NCT04676360
ALK-positive relapsed/refractory Neuroblastoma		Phase I/II	Ceritinib+ Ribocicliib			NCT02780128
ANBL1531: Children with Newly Diagnosed High-Risk Neuroblastoma		Phase III	131I-Metaiodobenzylguanidine (131I-MIBG) or Crizotinib Added to Intensive Therapy			NCT03126916
Relapsed ALK-positive lymphoma previously treated With ALK Inhibitors		Phase II	Lorlatinib			NCT03505554
ANHL12P1: newly diagnosed Stage II-IV ALCL		Phase II	Brentuximab Vedotin or Crizotinib in Combination with Chemotherapy		Results available for the Brentuximab arb	NCT01979536
HR-NBL2: High-Risk Neuroblastoma Study of SIOP-Europe-Neuroblastoma (SIOPEN)		Phase II	Lorlatinib			NCT04221035
NANT 2015-02: ALK-driven Relapsed or Refractory Neuroblastoma Phase I		Phase I	Lorlatinib			NCT03107988
Relapsing/Refractory ALK-altered Anaplastic Large Cell Lymphoma		Phase II	Nivolumab		Evaluation of response in patients with progressive disease (Cohort 1) or as consolidative immunotherapy in patients in complete remission after relapse (Cohort 2)	NCT03703050
ALK-altered ALCL, IMT or Other Solid Tumors (Briga-PED)		Phase I/II	Brigatinib		Phase I dose escalation in ALK-altered ALCL or ALK-altered solid tumors. Phase II Cohort B1: ALK-altered IMT Cohort B2: ALK-altered ALCL	NCT04925609
ALK Fusion-positive Solid or CNS Tumors (prior treatment has proven to be ineffective or from whom there is no curative standard treatment available)		Phase I/II	Alectinib			NCT04774718
Solid Tumors Harboring ALK, ROS1, or NTRK1-3 Rearrangements (TRIDENT-1)		Phase I/II	Repotrectinib			NCT03093116
Solid tumors harboring NTRK 1/2/3 (Trk A/B/C), ROS1, or ALK gene rearrangements (Fusions) (STARTRK-2) Phase I/II		Phase I/II	Entrectinib			NCT02568267
Relapsed or refractory Advanced Solid Tumors, Non-Hodgkin Lymphoma, or Histiocytic Disorders with ALK or ROS1 Genomic Alterations (A Pediatric MATCH Treatment Trial)		Phase II	Ensartinib			NCT03213652
Malignant Melanoma with ALK alterations		Phase II	Ensartinib			NCT 03420508
Patients with Advanced NSCLC and Other Solid Tumors Harboring ALK Rearrangement or Activating ALK Mutation (ALKOVE-1)		Phase I/II	NVL-655			NCT05384626

Abbreviations: *ACTG2* Actin Gamma 2, Smooth Muscle, *ALCL* anaplastic large cell lymphoma, *CAD* carbamoyl-phosphate synthetase 2, aspartate transcarbamylase, and dihydroorotase, *CCDC6* Coiled-Coil Domain Containing 6, *CR* complete response, *DCTN1* dynactin, *DLBCL* diffuse large B cell lymphoma, *DOR* duration of response, *EML4* echinoderm microtubule-associated protein like-4, *GBM* glioblastoma multiforme, *GOPC* Golgi Associated PDZ And Coiled-Coil Motif Containing, *IMT* inflammatory myofibroblastic tumor, *KANK1* KN Motif And Ankyrin Repeat Domains 1, *KIF4B/5B* kinesin family member 4B/5B, *NA* not available, *NPM1* nucleophosmin1, *NSCLC* non-small cell lung cancer, *PFS* progression free survival, *PR* partial response, *PPFBP1* PPFIA Binding Protein 1, *PRRC2B* proline rich coiled-coil 2B, *SD* stable disease, *SPECC1L* sperm antigen with calponin homology and coiled-coil domains 1 like, *SQSTM1* sequestosome 1, *STRN* striatin, *TFG* TRK-fused gene, *VCL* vinculin.

Butrynski and colleagues were one of the first to report a sustained partial response to crizotinib in a patient with *ALK*-rearranged IMT and mechanistically compared it with the absence of activity of crizotinib in another patient with IMT without *ALK* rearrangement^79^. Over the years, multiple basket trials have evaluated the efficacy of ALK inhibitors in tumors with *ALK* aberrations. The second pediatric strategy forum for ALK inhibition in pediatric malignancies provided an overview of the current status and future direction of ALK inhibitors in management of pediatric patients with ALCL, IMT and neuroblastoma^98^. The forum identified some key challenges of accruing patients suffering from these rare tumors in multiple clinical trials^98^.

A phase I/II children’s oncology group study of 26 *ALK*-altered ALCL and 14 IMT pediatric patients showed promising activity of crizotinib in these cancers. The overall response rate (ORR) in relapsed/refractory/unresectable IMT was around 86% and the ORR in *ALK*-altered ALCL was 83-90% (depending on dose). Complete response rate was 36% in IMT and over 80% in ALCL. Responses were durable^130^. Fukano et al reported results of a small phase II study of alectinib in 10 relapsed ALCL patients treated in Japan, which showed an impressive complete response rate of 60%- and 1-year overall survival rate of 70%^35^. A phase Ib study, PROFILE 1013 (NCT01121588) evaluated efficacy of crizotinib in 44 patients (≥15 years) suffering from *ALK*-altered ALCL, IMT and other malignancies. The ORR was 53% (95% confidence interval [CI], 28–77) for ALCL (mostly complete responses); 67% for IMTs (mostly PRs); and 12% (95% CI, 2–36) for other tumors, with two PRs in patients affected by colon carcinoma (lasting two years) and medullary thyroid cancer, respectively; an additional patient with neuroblastoma had stable disease for 19 months^132^. The median duration of treatment was almost three years for patients with ALCL and IMTs^132^. *ALK*-altered DLBCL is a rare subtype of large B cell lymphoma which resembles ALCL, with plasmablastic differentiation; one patient with *ALK*-altered DLBCL in this study had stable disease for almost four years with crizotinib^132^. The long-term follow up results of these studies have led to the FDA approval of crizotinib for *ALK*-altered ALCL and IMT^34,36^. Similar results have been observed in *ALK*-altered ALCL, IMT and some neuroblastoma cases treated ceritinib^143^. The ORR was ~75% (6/8) and 70% (7/10) among ALCL and IMT patients that harbor ALK rearrangements, respectively but it was only around 20% (6/30) in neuroblastoma (which usually harbors mutations in *ALK)*. Among neuroblastoma cases, response to ceritinib were observed in cases carrying ALK p.Arg1275 mutations. Because of the trial set-up, the exact type of ALK alteration was not always reported so it is unknown if there were specific *ALK* point mutations in neuroblastoma that were resistant to ceritinib.

Potential reasons for the attenuated clinical activity in some groups is due to ALK alterations other than fusions/rearrangements. Some of them are reported in (Table 4)^35,48,73,74,85,94,126,130–149^. For instance, *ALK* point mutations were permitted in some studies, and mutations such as F1174L (common *ALK*‐activating mutations seen in neuroblastoma) have been shown to confer resistance to crizotinib in neuroblastoma and other cancers^82^. Investigators believed that third generation TKI’s like lorlatinib could solve this problem and be more effective in counteracting these resistant mutations in neuroblastoma^98^. However, results of a recently concluded phase I neuroblastoma consortium study were largely unsatisfactory. In this study 33 relapsed/refractory patients aged 2-17 (cohort A1) and aged 15-50 (cohort A2) were presented in ASCO 2020. Lorlatinib was well tolerated, in cohort A2, 10% (1/10) patients attained CR and 30% (3/10) attained PR whereas only 5.5% (1/18) patients in cohort A1 had PR^148^. *MYCN* overexpression in neuroblastoma has also been associated with resistance to ALK inhibitors^82,83^.

*ALK* aberrations have also been described, albeit uncommonly, in rare hematologic disorders such as histiocytosis. *ALK*-aberrant histiocytosis usually harbors a *KIF5B-ALK* fusion, though other fusions are also observed^74^. Kemps et al reported retrospective data on 39 ALK-positive histiocytosis cases with 37 confirmed *ALK* rearrangements. They showed that advanced stage *ALK*-altered histiocytosis patients including those with neurological disease can be effectively treated with ALK inhibitors (11/11 objective and sustained response were achieved with ALK inhibitors such as crizotinib, alectinib, brigatinib and lorlatinib in first, second or further line setting^74^).

The use of crizotinib has also led to prolonged stabilization of disease in individual cases of recurrent glioblastoma^150^ but its utility in management of glioblastoma’s with ALK aberrations is questionable. For example, in the ASCEND 10 study^140^ none of the 12 ALK positive glioblastoma patients responded to crizotinib. Unfortunately, this study was terminated early and since enrollment was only based on ALK positive immunohistochemistry, there is no data on types of ALK alterations included in the glioblastoma cohort available in public domain. One plausible reason for suboptimal treatment response to crizotinib could be the higher prevalence of ALK mutations over fusions in this cancer cohort.

Resistance to first line *ALK* inhibition has also been seen in some cases which are often overcome by second or third line *ALK* inhibition. Second and third generation ALK inhibitors like alectinib, ceritinib, brigatinib and lorlatinib have good CNS penetration and are effective in management of crizotinib resistant and relapsed/refractory patients with CNS metastasis^151,152^. Alectinib is being studied in pediatric patients with ALK fusion-positive solid or CNS tumors (NCT04774718). Brigatinib is also being evaluated in a clinical trial in relapsed and refractory ALCL, IMT and other pediatric cancers (NCT04925609). We have also enlisted ongoing clinical trials of ALK inhibitors with their clinical trial.gov ID’s in (Table 4)^35,48,73,74,85,94,126,130–149^.

In a study of nine *ALK* -rearranged colorectal cancers (~0.2% of colorectal malignancies), one metastatic cecal cancer patient with a STRN*-ALK* fusion protein was treated with the ALK inhibitor ceritinib, which resulted in a marked decrease in size of a skin metastasis, and resolution of all contrast-enhancing tumors on imaging studies. This patient-derived treatment benefit for almost 9 months^49^. Some other studies and case series have reported responses to second and third-generation ALK inhibitors in colorectal cancer^141^, renal cell carcinoma^126^, pancreas adenocarcinoma^94^ and thyroid cancers^138,139^, with all tumors notably bearing fusions.

Results of CTO32 study were recently presented at a national meeting. In this study, 21 patients with various tumor types harboring *ALK* aberrations (10 patients with *ALK* gene rearrangement and 11 with *ALK* mutation or amplifications) were treated with alectinib. Among the 10 cancer patients with *ALK* rearrangements, three had partial responses (PRs). Interestingly, there were no responses among the 11 patients. with *ALK* mutations or amplification. The median progression-free survival (PFS) was 8.2 months (95% CI, 1.7–13.6) in patients with *ALK* rearrangements vs 1.8 months (95% CI 1.1–5.5) for those with *ALK* mutation or amplifications^153^. Results of subgroup F (cases harboring *ALK* fusions) of the NCI match study^145^ were recently reported as well. Two patients with colorectal cancer, one with carcinoma of unknown primary and one with leiomyosarcoma were included in this cohort and treated with crizotinib. At the time of analysis, the ORR was 50% with one complete response in the leiomyosarcoma patient and partial response in one colorectal cancer patient, again showing that tumors harboring *ALK* fusions/rearrangement are responsive to ALK inhibitors.

It is important to note that some of the above-mentioned studies solely relied on immunohistochemistry (IHC) to identify ALK overexpressing tumors and enrolled ALK-positive cancer patients based on the IHC findings. Next generation sequencing tests were less frequently utilized to identify ALK alterations, which could have affected the final treatment outcome in some cases. We believe that response to ALK inhibitors depends on the type of *ALK* alterations. For example*, ALK* point mutations and amplifications are common in neuroblastoma, whereas *ALK* fusions*/*rearrangements are more common in IMT and ALCL, but all of these tumor types can present with ALK overexpression. Based on data collected from some international studies that included multiple disease cohorts, we can conclude that, among cancer cohorts like IMT and ALCL known to have higher frequency of ALK rearrangements, ORR were much higher, ranging from 56 to 100%^35,130–132,140,143^ compared to 9 to 20%^131,143^ observed in cancer cohorts such as neuroblastoma and other tumors where *ALK* mutation and amplifications are generally more prevalent). Furthermore, anecdotal responses of ALK fusion/rearranged cancers across a broad array of malignancies and with multiple different ALK inhibitors have been reported in (Table 4)^35,48,73,74,85,94,126,130–149^.

Poor responses in some disease groups seem attributable mostly to the *ALK* alterations being mutations or amplifications rather than fusions/rearrangement, the fact that ALK overexpression does not always mean that there is a fusion/rearrangement present, and conceivably due to co-existing drivers. Because of the importance of fusions/rearrangements and their potential actionability, it has been suggested that sequencing RNA rather than DNA might be more effective in detecting ALK fusions/rearrangements, given the difficulties of covering all introns from which rearrangements can arise^154^.

Taken together, patients with a wide variety of common and rare solid and hematologic malignancies are responsive to any one of multiple ALK inhibitors, especially if their tumors bear fusions/rearrangements, with responses occurring less frequently with other types of *ALK* alterations.

## Tests to diagnose *Alk* alterations

Three main detection methods are utilized in clinical practice to detect *ALK* alterations. These include fluorescence in situ hybridization (FISH), immunohistochemistry (IHC) and next-generation sequencing (NGS) technologies. In a study by Bernicker et al. ^155^, real-world data of 41,728 patients with NSCLC diagnosed in community medical centers in the USA from January 2012 to May 2019 was evaluated to describe the ALK testing trend. The study showed that the ALK test use rates in eligible patients suffering from NSCLC dramatically rose from 59.5% in 2012 to 84.1% in the year 2019^156^. ALK testing rates have been higher in patients of younger age (<50 years), Asian race, non-squamous histology type, nonsmokers, and initial stage as stage IV.

FISH was the dominant method of ALK detection (81% of all testing) in the earlier part of this decade and is still considered a gold standard of ALK fusion detection, but NGS testing has quickly gained ground over the last five years. According to some estimates, by the first half of 2019, 45.99% of tests were performed by NGS compared with FISH, which was used in 37.68% of all cases^155^. Signal intensity variation and inter-observer variability have limited the use of FISH in a clinical setting. The cost-effectiveness and rapid turnaround time of IHC have made it an attractive choice for clinicians and pathologists. The accuracy of this technique is retained when combined with high-performance antibodies. The concordance rate between FISH and IHC was found to be around 80.6% in a large pooled metanalysis of 11,000 cases^157^. However, this means that substantial numbers of IHC patients do not have one of the highly actionably *ALK* fusions/rearrangements.

Another popular method of direct *ALK* fusion detection is reverse transcriptase PCR(RT-PCR) based testing. It has a rapid turnaround time and according to some estimates concordance rate between FISH and RT-PCR is around 89%^158^. Even so, NGS technology is quickly emerging as a preferred method for comprehensive testing in cancer. It allows broad coverage of genomic regions of interest, fusion partner characterization and improves detection of relevant genomic alterations, and most importantly, permits hundreds of genes to be interrogated with a single test. At the DNA level, it is sometimes difficult to detect gene-fusion expression, particularly if breakpoints involve long intronic regions that may not be covered by hybridization-capture probes. To address this critical issue, targeted RNA-based NGS assays have been developed which are more sensitive and effective in detecting gene fusions^154,159^. Overall, NGS testing has the best throughput among all these testing modalities.

## Mechanisms of resistance to ALK inhibitors

Several mechanisms of ALK resistance have been proposed. These include the resistance mutations, differential sensitivity to different ALK inhibitors, type of alteration (with fusions/rearrangements being more sensitive to ALK inhibitors that mutations/amplifications and co-existent molecular drivers).

**Fig. 2:**
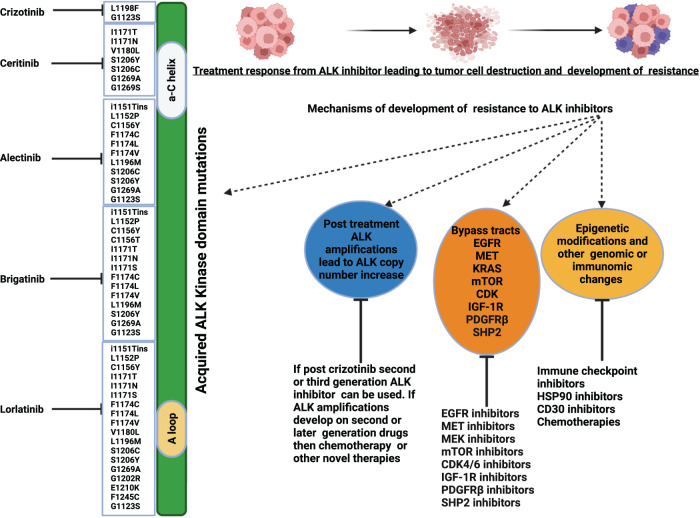
Examples of Resistance Mechanisms and Treatment Strategies for ALK inhibitor resistant tumors. This figure Illustrates mechanisms of development of resistance to ALK inhibitors and possible management strategies. For example, development of bypass pathways like EGFR, MET, PDGFR, IGF-1R, CDK, mTOR and SHP2 can lead to resistance and can be targeted by their respective inhibitors. Resistance can also develop because of epigenetic modifications and other immunomic changes in tumor, which may theoretically be targeted by heat shock protein (HSP) 90 inhibitors, Cluster differentiation (CD) 30 inhibitors and immune check point inhibitors. Similarly, formation of *ALK* amplifications may also be a mechanism of resistance and can be treated with chemotherapy. This figure also illustrates various acquired tyrosine kinase domain mutations which can be targeted by different generations of ALK inhibitors. The mutations listed on the left of ALK kinase domain are sensitive to respective ALK tyrosine kinase inhibitors. For example, crizotinib inhibits G1123S, L1198F resistant mutation. Ceritinib inhibits I1171T, I1171N, V1180L, S1206C, S1206Y, G1269A. Alectinib inhibits i1151Tins, G1123S, L1152P, C1156Y, F1174C, F1174L, F1174V, L1196M, S1206C, S1206Y, G1269A resistant mutations. Brigatinib inhibits i1151Tins, L1152P, C1156Y, I1171T, I1171N, I1171S, F1174C, F1174L, F1174V, V1180L, L1196M, S1206Y, G1269A and Lorlatinib inhibits i1151Tins L1152P, C1156Y, I1171T, I1171N, I1171S, F1174C, F1174L, F1174V, V1180L, L1196M, S1206C, S1206Y, E1210K, G1269A, E1210K, G1202R, E1210K, F1245C. Created by Biorender.com.

The development of secondary resistance mutations in the ALK kinase domain, can be a resistance mechanism^160^. For instance, L1196M mutation corresponds to a gatekeeper residue, a residue located in the ATP-binding pocket of a protein kinase that, when mutated, causes a change in the structure of the kinase that prevents ALK inhibitor binding. Certain resistance mutations affect residues adjacent to the N-terminus for example (C1156Y, L1152R, and I1151Tins) and C-terminus of the αC helix for example (F1174C/L/V)^71^. These mutations enhance the kinase’s ATP-binding affinity and increase its enzymatic activity. G1202R, D1203N, and S1206Y/C represent another class of solvent front *ALK* resistance mutations that impair drug binding likely through steric hindrance^161^. Earlier studies have shown that G1202R confers high-level resistance to first- and second-generation ALK inhibitors and is susceptible to third generation ALK inhibitors like lorlatinib^71,160,161^. Differential sensitivity to ALK mutations between different ALK inhibitors has also been reported in Fig. 2^25,71,115,161–170^. For example, crizotinib^161^ can inhibit G1123S mutations which is resistant to ceritinib. Some cell lines harboring I1171N and I1171T mutations that are resistant to alectinib but are sensitive to ceritinib^161,168^. Conversely, L1152P, L1152R, F1174 C, F1174 L and F1174V mutations confer resistance to ceritinib^161,168^ but are sensitive to alectinib, brigatinib and lorlatinib. Lorlatinib is effective against mutations like F1245C, E1210K and G1202R which confer resistance to second generation ALK inhibitors. Sequential treatment with ALK inhibitors and exposure to third generation agents such as lorlatinib can also lead to development of resistant compound mutations such as ALK L1198F/C1156Y and G1269A/I1171S^161^. Tumors harboring these mutations can sometimes be rechallenged with first or second generation ALK inhibitors like crizotinib^167^ and ceritinib^171^ respectively. Some other highly resistant compound mutations such as G1202R/L1196M and D1203N/I1171N have also been reported that do not respond to first, second or third generation ALK inhibitors. A study by Dagogo-Jack et al. reported cooccurrence of ALK D1203N mutation in exon 23 with the I1171N exon 22 mutation in some lorlatinib-resistant cases. The allele frequencies of the two ALK-resistant mutations suggested that they were likely in cis, but the allelic configuration could not be confirmed as the two exons were separated by approximately 1600 base pairs^172^. Another study by Recondo et al. also reported the presence of a D1203N mutation, present in “cis” with the L1196M mutation in lorlatinib-resistant cases^173^. Post-treatment ALK amplifications occur less frequently than secondary mutations, but are also a recognized cause of acquired resistance to crizotinib^161^.

Apart from the above-mentioned ALK inhibitor-dependent resistant mechanisms, there are ALK inhibitor independent resistance pathways. As an example, epigenetic modifications and epithelial-mesenchymal transitions can lead to ALK-independent resistance^174^ as can aberrant activation of alternate kinases that promote ALK-independent growth through bypass pathways, including via the epidermal growth factor receptor (EGFR), human epidermal growth factor receptor type 2(HER2), and insulin-like growth factor-1(IGF-1R) receptor signals^23,175^ as well as platelet derived growth factor receptor beta (PDGFRβ), mammalian target of rapamycin (mTOR), Mesenchymal Epithelial Transition (MET) and others^71^. Activation of the MEK pathway can also contribute to ALK inhibitor resistance, through deficiency/low expression of Wiskott–Aldrich syndrome protein (WASP) protein^176^. Interestingly, activation of Interleukin (IL)-10RA signaling pathways also contribute to first generation ALK inhibitors resistance. IL-10 is a known immunosuppressive factor that, promotes T cell exhaustion, inhibits effector T cells and T cell activation^177^. Finally, high expression of P-glycoprotein (P-gp), an ATP-dependent efflux pump which is encoded by the multidrug resistance 1 (MDR1) gene can also lead to ALK independent resistance. It has been reported to be a potential cause of drug resistance and reduced CNS penetration of crizotinib and ceritinib^71^. Genome-wide sequencing studies have shown that protein tyrosine phosphatases such as PTPN1 and PTPN2 are involved in ALK inhibitor resistance in lymphoma. *PTPN1* and *PTPN2* genes regulate ALK and Src homology region 2 -containing protein tyrosine phosphatase 2 (SHP2) phosphorylation. Based on these findings, it can be postulated that combined inhibition of ALK and SHP2 is an approach that merits study in ALCL^178^. Prokoph et al. also reported that resistance to crizotinib in ALCL cases can be driven by upregulation of interleukin-10 receptor subunit alpha (IL10RA). The elevation of IL10RA expression rewires the signal transducer and activator of transcription 3 (STAT3) molecular signaling pathway which bypasses otherwise critical phosphorylation induced by NPM1-ALK fusion^10^. Figure 2^25,71,115,161–170^ illustrates some of the common resistant mechanisms seen with ALK inhibition.

### Targeting resistance with novel agents and paradigms

Resistance mutations acquired on treatment with an ALK inhibitor can sometimes be overcome by switching to a newer generation ALK inhibitor (Fig. 2)^25,71,115,161–170^. Earlier studies have shown that long-term ALK inhibition was also associated with enhanced IGF-1R signaling, which leads to the development of resistance pathways. IGF-1R increases the phosphorylation of ALK and its downstream effectors in ALK-aberrant ALCL cells. It further promotes the survival of these cancer cells by increasing the expression of anti-apoptotic proteins like Bcl-2 and Bcl-xl^179^. IGF-1R inhibitors sensitize tumor cells to the effects of ALK inhibition. Hence, co-targeting ALK and IGF-1R can improve treatment response in ALK inhibitor naïve setting and can reverse resistance in ALK inhibitor exposed setting. Ceritinib is a second-generation ALK inhibitor which is also a potent IGF-1R inhibitor^25^. This off-target effect of ceritinib might explain, in part, its efficacy in crizotinib-resistant tumors. Unfortunately, target kinase domain alterations and bypass signaling do not appear to be mutually exclusive and can further lead to resistance from second and third generation ALK inhibitors^71^.

In those unusual cases where there are co-existent *ALK* and *EGFR* alterations, which occurs in a subset of NSCLC, dual inhibition of EGFR and ALK can be a useful strategy in treating patients that develop resistance to ALK inhibitors such as crizotinib, ceritinib, and alectinib. Investigators have shown that afatinib may be a promising treatment for overcoming ceritinib resistance in ALK-positive NSCLC cells by inhibiting the EGFR^162^ and neuroregulatory protein (NRG1) signaling^180^ pathways. Interestingly brigatinib has in vitro activity against both *EGFR* and *ALK* alterations and can be used to target EGFR induced resistance to ALK inhibitors^181^. The mTOR pathway also acts as a bypass pathway of resistance and some investigators have shown a synergistic effect of combining ALK inhibitors with mTOR inhibitors in lymphoma cells^165^. There is also evolving data to show that increased expression of hepatocyte growth factor (HGF) and MET amplification can lead to resistance from some ALK inhibitors. This can be mitigated by combining metformin and second-generation ALK inhibitors such as alectinib^182^ or perhaps by adding a MET inhibitor^183^.

A preclinical study by Wood et al. showed that dual inhibition with a CDK4/6 inhibitor like ribociclib and ALK inhibitor such as ceritinib can lead to complete regression of *ALK*-aberrant neuroblastoma xenograft tumors^164^. This combination is now being investigated in a phase I Next Generation Personalized Neuroblastoma Therapy (NEPENTHE) study (NCT02780128). NPM-ALK-altered ALCL cells are associated with high expression of JUNB, JUN, PDGFRα and PDGFRβ mRNA and protein. Some preclinical studies and case reports have shown that therapeutic blockade of PDGFRβ with agents like imatinib in combination with ALK inhibitors can lead to significant reduction in size of tumor mass and alleviate relapse of ALCLs after exposure to single agent ALK inhibitors^163^.

A customized (N-of-1) combinatorial targeted therapy-based approach can also be utilized to tackle resistant mechanisms^184–187^. The efficacy and safety of NGS-informed customized combination therapy was reported in the analysis of the I-PREDICT studies in a variety of cancers. Targeting a larger fraction of known molecular alteration with a customized multidrug regimen leads to better disease control rates, higher response rates and longer PFS and survival than targeting fewer molecular alterations^184–187^.

## Case presentation

**Fig. 3:**
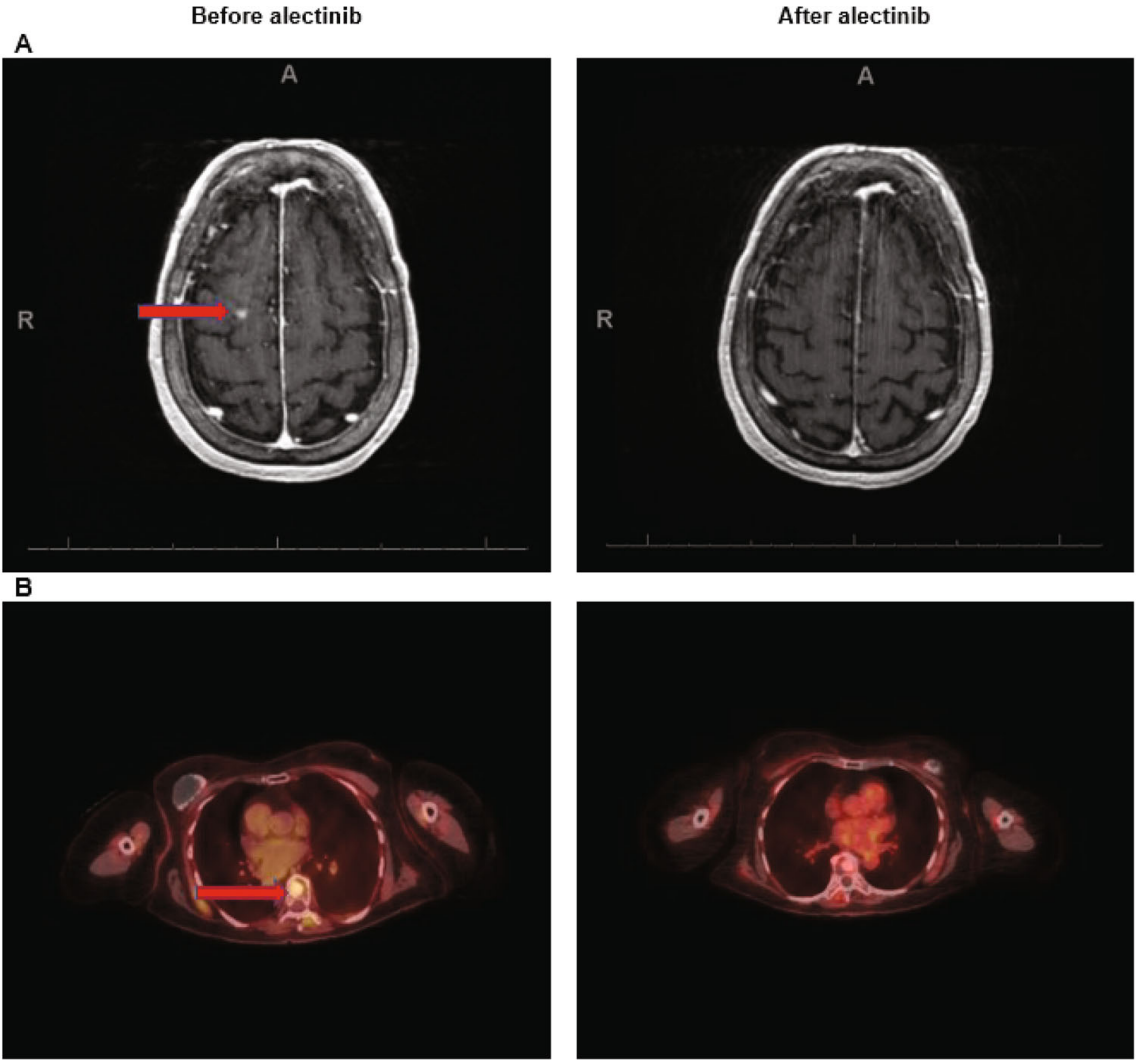
Case of 73-year-old woman with Erdheim Chester disease (non-Langerhans histiocytosis) and ALK-KIF4B fusion responds to alectinib. Figure showing response to alectinib in follow up scans taken 2 months after treatment initiation. **A** MRI brain showing resolution of the lesion in the right superior frontal gyrus (arrow). **B** PET/CT showing decreased FDG uptake in T8 sclerotic lesion. Red arrows show lesions in the before treatment scans.

A 73-year-old woman with widespread Erdheim-Chester disease (ECD) (non-Langerhans histiocytosis) with an *ALK-KIF4B* fusion, who had a remarkable response to alectinib is discussed. This patient had a history of breast cancer and initially presented with right knee pain. Imaging revealed widespread osseous, extraosseous deposits and leptomeningeal disease. Biopsy of left cervical lymph node indicated histiocytic proliferation compatible ECD (negative for *BRAFV600E* mutation). Additional molecular testing with targeted NGS of tumor tissue and plasma derived circulating tumor DNA showed *KIT M541L* mutation (tissue) and *TP53* mutations (C135W and C277Y) (blood biopsy). Earlier studies have shown that alterations in the MAPK pathway like MAP2K1, MAP2K2, GNAS, NF1, and RAS mutations are quite common in BRAF wildtype ECD and can be targeted by MEK inhibitors such as cobimetinib or trametinib, albeit with a need for reduced doses in patients with ECD^188,189^. The patient was treated with trametinib 1–1.5 mg orally daily, and imaging with PET/CT and MRI demonstrated partial response. Unfortunately, she developed cardiac complications, which precluded trametinib continuation. Because studies have demonstrated a role of proinflammatory cytokines such as interleukin-1 and -6 in the pathogenesis of ECD^190^ and Killu AM and colleagues had also reported a case of ECD patient with cardiac involvement, who was successfully treated with an IL-1 receptor antagonist^191^, she was, switched to the IL-1 receptor antagonist anakinra, which she tolerated well and had stable disease for 18 months. Patient later consented to the Institutional Review Board (IRB) approved MD Anderson Cancer Center (MDACC) protocol number RC04-567. Additional testing for gene fusions performed on archival tissue blocks revealed an *ALK-KIF4B* fusion. Therefore, an ALK inhibitor-- alectinib 600 mg PO daily-- was added to her treatment regimen. Follow up PET/CT and MRI after 2 months demonstrated remarkable improvement in brain lesions (Fig. 3). She continues to do well six months after starting alectinib without major toxicity.

### In summary

ALK is now an established therapeutic target in NSCLC and several other hematologic and solid malignancies. *ALK* alterations include mutations, amplifications, overexpression by IHC, and fusions/rearrangements. Multiple ALK inhibitors have entered the clinic; to date, five of them—alectinib, brigatinib, ceritinib, crizotinib, and lorlatinib-- have become standard therapies for advanced *ALK*-aberrant NSCLCs. Crizotinib is also FDA approved for *ALK*-aberrant IMT and ALCL, including in children, reflecting activity of these agents in both solid and hematologic malignancies and across the age spectrum (Table 1)^28–34,36–39^. Importantly, the regulatory authorizations for ALK inhibitors are for neoplasms bearing *ALK* fusions/rearrangements and, in those conditions, response rates are usually in the range of 50–85%. Although ALK fusions/rearrangements occur in ~3–7% of NSCLCs, ~50% of IMTs and 50–80% of ALCLs, such alterations are ultra-rare in other cancers, being found in only ~0.2% of patients, making studies in these diseases challenging, and perhaps below the feasibility threshold of prospective treatment trials, even those that are large scale such as NCI-MATCH and similar efforts^192,193^. Importantly, the literature is replete with reports of exceptional responses of multiple tumor types bearing *ALK* fusions/rearrangements, both solid and hematologic, including but not limited to colorectal cancer, histiocytosis, leiomyosarcoma, lymphoma, multiple myeloma, neuroendocrine cancer, ovarian cancer, pancreatic cancer, renal cancer, thyroid cancer (even the aggressive anaplastic variant) after the patient was given an ALK inhibitor. Most of these reports used crizotinib or alectinib, but each of the approved ALK inhibitors have demonstrated efficacy in some of the published studies (Table 4)^35,48,73,74,85,94,126,130–149^. Responses to ALK inhibitors can also be observed in diseases such as neuroblastoma, which bear *ALK* mutations (rather than fusions/rearrangements), but the response rates are generally only in the ~10–20% range. Based on current data, it seems reasonable to posit that ALK inhibitors have tissue-agnostic activity in malignancies bearing *ALK* fusions/rearrangements.

### Reporting summary

Further information on research design is available in the Nature Research Reporting Summary linked to this article.

### Supplementary information

Supplementary Table 1

Reporting Summary

## Data Availability

We have not analyzed any publicly available databases for this article. We have appropriated cited all clinical data included in this review article.
