# Supplementary material for: ALK fusions in the pan-cancer setting: another tumor-agnostic target?
Source: NPJ Precis Oncol. 2023 Sep 29;7:101. doi: 10.1038/s41698-023-00449-x (PMC10542332; doi:10.1038/s41698-023-00449-x)
Supplement: Supplementary file 1 — Supplementary Table 1 [file 41698_2023_449_MOESM1_ESM.pdf]

Supplemental Table 1. Examples of key studies of targeted therapies in ALK-altered NSCLC (see Table 4 for ALK-altered cancers other than NSCLC)

ALK inhibitors	FDA approval	Control arm	Line of treatment	Examples of targets (IC50) Selleckchem.com	Clinical indications	Median PFS	Clinical trials
Crizotinib	2011<br>Accelerated approval	No control arm in PROFILE 1005	Second	ALK (24nm), ROS1 (<0.025), and c-MET (11nm)	Unresectable/<br>metastatic disease ALK-altered NSCLC after failure of ≥1<br>lines of treatment	8.1 months	PROFILE1005 [111] (Phase II)
Crizotinib		Platinum +pemetrexed (in PROFILE 1014)	First	ALK (24nm), ROS1 (<0.025), and c-MET (11nm)	Unresectable/<br>metastatic NSCLC (ALK-altered) chemotherapy naïve	10.9 vs 7 months	PROFILE 1014(Phase III) [113]
Crizotinib	2013<br>Full approval	Docetaxel/pemetrexed	Second	ALK (24nm), ROS1(<0.025), and c-MET (11nm)	unresectable/<br>metastatic NSCLC (ALK-altered) previously treated with platinum-based chemotherapy	7.7 vs 3 months	PROFILE 1007(Phase III) [114]
Ceritinib	2017	Platinum	First	ALK(0.2nM), IGF-1R (8nM), InsR (7nM), and STK22D (23nM)	Unresectable/<br>metastatic NSCLC with ALK-altered tumors	16.6 vs 8.1 months	ASCEND 4(Phase III) [118]
Ceritinib	2014	Single-arm studies	Second	ALK(0.2nM), IGF-1R (8nM), InsR (7nM), and STK22D (23nM)	unresectable/<br>metastatic NSCLC (ALK-altered) prior exposure to crizotinib	6.9 months in patients who had prior exposure to crizotinib in ASCEND 1	ASCEND 1 (Phase I) [119]<br>ASCEND 2 (Phase II) [122]
Ceritinib		Pemetrexed or docetaxel	Second	ALK, IGF-1R, InsR, and ROS1	Unresectable/<br>metastatic NSCLC (ALK-altered) that have progressed on chemotherapy and crizotinib	5.4 vs 1.6 months	ASCEND 5(Phase III) [116]
Ensartinib	Approved in First line in China	Crizotinib	First	MET, Axl, ABL, EPHA2, LTK, ROS1, and SLK	Unresectable/<br>metastatic NSCLC (ALK-altered)	25.8 vs 12.7 months	eXalt3 (Phase III) [123]
Brigatinib	2020	Crizotinib	First	ALK (0.6 nM) and ROS1 (0.9 nM), also inhibits IGF-1R, and FLT-3 as well as EGFR deletion and point mutations.	ALK-altered unresectable/<br>metastatic NSCLC	24 vs 11 months	ALTA-1L(Phase III) [121]
Brigatinib<br>Oral brigatinib 90 mg once daily (arm A) or 180 mg once daily (arm B)	2017	Single arm study	Second	ALK (0.6 nM) and ROS1 (0.9 nM), also inhibits IGF-1R, and FLT-3 as well as EGFR deletion and point mutations.	Crizotinib-pretreated unresectable/<br>metastatic ALK-altered NSCLC patients	9.2months in arm A and 16.7 months in arm B	ALTA (Phase II) [124]
Alectinib	2017	Crizotinib	First	ALK (1.9nM) and RET (4.8nM) with CNS activity.	ALK-altered unresectable/<br>metastatic NSCLC	34.8 vs 10.9 months	ALEX (Phase III) [120]
Alectinib	2015	Single arm study	Second	ALK(1.9nM) and RET (4.8nm) with CNS activity.	Crizotinib-pretreated or intolerant unresectable/<br>metastatic NSCLC	8.1 months	NP28761 (NCT01871805) [115]
Alectinib		Pemetrexed or Docetaxel	Second	ALK(1.9nM) and RET (4.8nm) with CNS activity.	Crizotinib-pretreated or intolerant unresectable/<br>metastatic NSCLC	7.1months vs 1.6 months	ALUR (Phase III)[127]
Lorlatinib<br>(3rd generation)		Crizotinib	First	ALK wild type (<0.07 nM), ROS1(<0.02 nM), ALK (L1196M) (0.7nM) as well as inhibits TYK1, FER, FPS, TRKA, TRKB, TRKC, FAK, FAK2, and ACK	ALK-altered unresectable/<br>metastatic NSCLC<br>ALK inhibitor naive	Not evaluable vs 9.3 months	CROWN (Phase III) [117]
Lorlatinib<br>(3rd generation)	2018	Single arm study	Second/Third	ALK wild type (<0.07 nM), ROS1(<0.02 nM), ALK (L1196M) (0.7nM) as well as inhibits TYK1, FER, FPS, TRKA, TRKB, TRKC, FAK, FAK2, and ACK	ALK-altered unresectable/<br>metastatic NSCLC whose disease progressed on crizotinib and at least one other ALK inhibitor or alectinib or ceritinib used as the first ALK inhibitor	Not reported	B7461001 (Phase II)<br>[112, 125]
Entrectinib<br>FDA approved in 2019 for NTRK fusions and ROS1 fusions only	No	Single arm study	First-line	(Trk) A/B/C, ROS1, NTRK, and ALK	Solid tumors with rearrangements of the TRK family, ROS1, or ALK.	Median PFS of 8.3 months in 7/27 ALK naïve patients in these 2 studies	Alka-372-001 STARTRK-1<br>[126]

Abbreviations: anaplastic lymphoma kinase tyrosine kinase inhibitors (ALK INHIBITORS); central nervous system (CNS); epidermal growth factor receptor (EGFR); mesenchymal-epithelial transition factor (MET); non-small-cell lung cancer (NSCLC); neurotrophic tyrosine receptor kinase (NTRK); the insulin-like growth factor-1 receptor (IGF1R); rearranged during transfection (RET); c-ros oncogene 1 (ROS1); tropomyosin receptor kinases A/B/C (Trk A/B/C).
